# A new era in sports science: the launch of *BMC Sports Science, Medicine and Rehabilitation*

**DOI:** 10.1186/2052-1847-5-1

**Published:** 2013-03-28

**Authors:** Elizabeth C Moylan, Genevieve Horne

**Affiliations:** 1 BioMed Central Ltd, 236 Gray’s Inn Road, London, WC1X 8HL, UK

## Abstract

This Editorial celebrates the launch of *BMC Sports Science, Medicine and Rehabilitation* within the *BMC* series of journals published by BioMed Central. *BMC Sports Science, Medicine and Rehabilitation* incorporates the recently closed *Sports Medicine, Arthroscopy, Rehabilitation, Therapy & Technology* (*SMARTT*) with an expanded scope and Editorial Board. *BMC Sports Science, Medicine and Rehabilitation* will fill its own niche in the *BMC* series alongside other companion journals including *BMC Physiology, BMC Musculoskeletal Disorders* and *BMC Surgery.*

## Editorial

This month sees the launch of the new journal *BMC Sports Science, Medicine and Rehabilitation*, which considers articles on all aspects of human exercise physiology, sports and exercise medicine, including rehabilitation, traumatology, cardiology, physiology and nutrition. The new journal also incorporates *Sports Medicine, Arthroscopy, Rehabilitation, Therapy & Technology* (*SMARTT*), which was previously under the stewardship of its Editors-in-Chief, Kai-Ming Chan and Masahiro Kurosaka. *SMARTT* was originally launched in 2009 as the official journal of the Asia–Pacific Orthopaedic Society for Sports Medicine (APOSSM) and was affiliated with the Japanese Orthopaedic Society of Knee, Arthroscopy and Sports Medicine (JOSKAS) [1]. At the end of 2012, the society decided to launch a separate journal and so BioMed Central has now incorporated *SMARTT* into *BMC Sports Science, Medicine and Rehabilitation*. The new journal sits comfortably within the *BMC* series of journals, which have become well-recognised in the research communities they serve.

*BMC Sports Science, Medicine and Rehabilitation* will maintain *SMARTT*’s ethos and broad scope but will not be affiliated with a particular society. The subject areas we will cover include physical activity and health, human nutrition, human exercise physiology, sports medicine and rehabilitation, and sport and exercise performance. Topics include (but are not limited to) surgery, traumatology, biomechanics, and injury prevention, and we have also broadened the scope of the journal to explicitly cover sports genomics, psychology, training and responses to this, cardiology and regenerative medicine.

The field of sports science and medicine encompasses a wide variety of disciplines. It seeks not only to improve levels of mental and physical fitness and performance, but also aims to further advance the treatment and prevention of injuries related to sports and exercise, along with improving overall health and nutrition. With the prevalence of obesity rising on a worldwide scale and public health policy aimed at increasing levels of exercise at a population level, the growth of sports science and medicine as field of research is going to be inevitable. As a Publisher we appreciate the need for a broad-scope sports science and medicine journal whilst ensuring that there is a strong focus on exercise medicine, physiology, and rehabilitation, as these are growing areas of research [2]. In publishing *BMC Sports Science, Medicine and Rehabilitation* we aim to capture these key areas and in collaboration with our Editorial Board, build a strong presence as a home for this research. *BMC Sports Science, Medicine and Rehabilitation* is a timely addition to our cluster of related BMC series journals.

As with the other journals in the *BMC*-series stable, *BMC Sports Science, Medicine and Rehabilitation* has an international Editorial Board that retains many of the *SMARTT* Editorial Board Members with additional new faces and comprises Section Editors, Associate Editors and Editorial Advisors. We are delighted that Mike Carmont will continue his strong involvement with the journal as a Section Editor and he provides his personal perspective on sports traumatology research in an accompanying 'Question and Answer' piece [3].

All previous *SMARTT* content will remain open access and will be freely accessible from the new *BMC Sports Science, Medicine and Rehabilitation* platform.

*BMC Sports Science, Medicine and Rehabilitation* will adopt an open peer review policy as is currently the case on all medical titles within the *BMC* series [4]. There are two levels to this ‘openness’. The first is that authors will be able to see who reviewed their manuscript; the second is that the reading public will also see who reviewed the article and how the authors responded, if the article is published. The reports and authors’ response will be available as part of the pre-publication history of the published article.

Research into the effect of open peer review has found numerous benefits, the most important of which are accountability, fairness, and giving credit to reviewers for their efforts [5-7]. However, we recognise that there are negatives also. Sometimes reviewers may feel uncomfortable signing a critical report, especially when recommending rejection [8]. The reluctance to open review also means that more potential peer reviewers have to be invited to review a manuscript openly than under a peer review system which is closed [Parkin EC *et al.* unpublished observations; [8-10]. We will report back on our findings with open peer review on *BMC Sports Science, Medicine and Rehabilitation* in the next two years.

We are really looking forward to working with our authors, peer reviewers and Editorial Board Members on the new *BMC Sports Science, Medicine and Rehabilitation* journal in the coming months. While the focus of the journal is to publish original sound research, we also welcome commentaries and correspondence that explore the expanding boundaries of this research field. We do hope you will take the time to visit the website and consider us for your future submissions.

## Competing interests

Both authors are employees of BioMed Central.

## Authors’ contributions

Both authors have been working on *BMC Sports Science, Medicine and Rehabilitation* and have contributed to this editorial. Both authors have read and approved the final text.

## Pre-publication history

The pre-publication history for this paper can be accessed here:

http://www.biomedcentral.com/2052-1847/5/1/prepub
